# World Health

**Published:** 1920-08-14

**Authors:** 


### World Health.

### A SCHEME OF FAR-REACHING IMPORTANCE.

We are now at the beginning of a world organisation for the control of health. Some time ago we gave a summary of the scheme submitted to the Council of the League of Nations for the constitution of an international health office, and the Council last week approved of the plan which will undoubtedly be adopted by the Assembly of the League next November. This means, in the first place, the organised co-operation of at least forty States of the world in the interests of health, and it is one of those non-political aspects of life towards which no nations who are still outside the League are likely to be unfriendly. It is a great task which will need careful, assiduous, and expert organisation. Like every other task of an international, as well as of a national, character, it needs the intelligent backing of all whose support counts for anything, and the first desideratum for this is a national health sense. It can, in any case, give the world a lead by expert investigation, advice, and assistance.

The International Health Office will consist of a general committee, delegates appointed by members of the League, together with delegates appointed by countries not members of the League, but represented on the committee of the Bureau of International Public Health. This committee meets at least once a year. There will also be a permanent committee, consisting of delegates of members of the Council, four members elected by the general committee, the president of the general committee, a representative of the League of Red Cross Societies, and a representative of the Governing Body of the International Labour Office.

It will be the special task of the permanent committee to draw up or draft new conventions or revise the existing one. Such conventions m[illegible] be submitted to the general committee for conside[illegible] tion and approval. A majority of two-thirds of [illegible] votes of the general committee will be necessary [illegible] adoption. There will be an International He[illegible] Bureau consisting of an international staff unde[illegible] medical secretary, who will be the most experien[illegible] public-health administrator obtainable in the wo[illegible] The chairman and members of the permanent co[illegible] mittee hold their appointments for three years o[illegible] but are eligible for re-election.

The functions of the organisation are:

1. To advise the League on health matters.

2. To co-ordinate national administrative hea[illegible] authorities.

3. To organise rapid interchange of informa[illegible] on epidemic diseases and simplify rapid actio[illegible] such information where it affects more than [illegible] country.

4. To be prepared to initiate or revise neces[illegible] international agreements on administrative ac[illegible] in health matters.

5. To co-operate with the international la[illegible] organisation for the protection of workers ag[illegible] sickness and injury arising out of employm[illegible] (This is most essential for checking the sprea[illegible] such diseases as phosphorus poisoning, [illegible] poisoning, anthrax, and the like. For co-oper[illegible] in these matters a labour representative is to s[illegible] the permanent committee.)

6. To co-operate with the League of Red C[illegible] Societies, thus assisting in checking world-[illegible] epidemics.

7. To advise other authorised voluntary org[illegible] sations.

THE PUBLIC WELL-BEING—(*continued*).

8. To organise health commissions at the request of the League.

The expenses will be borne by the League. It will be seen that the League of Red Cross Societies, which is playing so large a part in the endeavour to check the spread of typhus in Eastern Europe, has a conspicuous share in the scheme, and the medical correspondent of *The Times*, in welcoming the organisation, says that to prevent the preventable diseases now raging in Europe is a task demanding a degree of courage and persistence far above the average.

